# Serum galactomannan index for early prediction of mortality in immunocompromised children with invasive pulmonary aspergillosis

**DOI:** 10.1186/s12879-015-1014-9

**Published:** 2015-07-14

**Authors:** Seung Beom Han, Seong koo Kim, Jae Wook Lee, Jong-Seo Yoon, Nack-Gyun Chung, Bin Cho, Dae Chul Jeong, Jin Han Kang, Hack-Ki Kim, Dong-Gun Lee, Hyun Sil Lee, Soo Ah Im

**Affiliations:** Department of Pediatrics, College of Medicine, The Catholic University of Korea, Seoul, Republic of Korea; The Vaccine Bio Research Institute, College of Medicine, The Catholic University of Korea, Seoul, Republic of Korea; The Catholic Blood and Marrow Transplantation Center, College of Medicine, The Catholic University of Korea, Seoul, Republic of Korea; Division of Infectious Diseases, Department of Internal Medicine, College of Medicine, The Catholic University of Korea, Seoul, Republic of Korea; Department of Radiology, College of Medicine, The Catholic University of Korea, Seoul, Republic of Korea; Department of Pediatrics, Seoul St. Mary’s Hospital, 222, Banpo-daero, Seocho-gu, Seoul 137-701 Republic of Korea

**Keywords:** Invasive pulmonary aspergillosis, Galactomannan, Prognosis, Immunocompromised host, Child

## Abstract

**Background:**

Invasive pulmonary aspergillosis (IPA) is the most common invasive fungal disease in immunocompromised patients, and it has a 30 % mortality rate despite appropriate antifungal therapy. This retrospective study was performed to determine risk factors for mortality in immunocompromised children with IPA.

**Methods:**

Medical records of 45 probable/proven IPA cases diagnosed in children with hematologic/oncologic diseases were reviewed. Selected cases were divided into the survival (*n* = 30) and fatality (*n* = 15) groups based on survival at 12 weeks after antifungal therapy. Clinical characteristics and serum galactomannan indices (GMIs) were compared between the two groups.

**Results:**

Significantly more children in the fatality group were male (*p* = 0.044), not in complete remission of the underlying malignancies (*p* = 0.016), and had received re-induction/salvage or palliative chemotherapy (*p* = 0.035) than those in the survival group. However, none of these factors was significantly associated with mortality in a multivariate analysis. Serum GMIs were higher in the fatality group than in the survival group during the entire period of antifungal therapy, and serum GMI at 1 week after antifungal therapy was most significantly associated with mortality. A serum GMI > 1.50 at 1 week after antifungal therapy exhibited a sensitivity and specificity of 61.5 % and 89.3 %, respectively, in predicting mortality within 12 weeks after antifungal therapy.

**Conclusions:**

Higher serum GMI in the early phase of antifungal therapy was associated with mortality in immunocompromised children with IPA. These children should receive more intensive care for IPA than others.

## Background

The occurrence of invasive fungal disease (IFD) has been increasing in children with hematologic/oncologic diseases, and invasive aspergillosis (IA) became the most common form of IFD after the introduction of fluconazole prophylaxis [1–4]. Considering that most cases of IA are invasive pulmonary aspergillosis (IPA), IPA is the most common form of IFD in children with hematologic/oncologic diseases [5–7]. Because IPA causes a 25–35 % mortality despite the introduction of new antifungal agents such as voriconazole and echinocandins [5, 8–10], better efforts are necessary in order to improve outcomes of immunocompromised children with IPA. Predicting patients at high risk for mortality among immunocompromised children with IPA and providing more intensive therapy for such patients can be an effective strategy to reduce mortality in patients with IPA.

Serum galactomannan index (GMI) has been used as a criterion for IPA diagnosis [11, 12], and its value has been reported in both immunocompromised adults and children [13]. Mortality in IPA patients with persistently elevated serum GMI was reportedly higher than in those with negatively converted serum GMI during antifungal therapy [14–17]. Therefore, serum GMI may be useful to predict prognosis in immunocompromised patients with IPA. However, most of the studies that reported an association between serum GMI and prognosis in IPA patients enrolled both adults and children [16–20], and studies including only children are scarce [21].

Hence, this study was conducted to determine clinical or laboratory findings associated with mortality in immunocompromised children diagnosed with IPA. The results of the present study can help determine the risk for mortality among immunocompromised children with IPA and contribute to improve outcomes in such high-risk patients by providing more intensive therapy in the early phase of IPA.

## Methods

### Study design and patients

Medical records of children (<20 years of age) with hematologic/oncologic diseases diagnosed with IPA between April 2009 and March 2014 at Seoul St. Mary’s Hospital, College of Medicine, The Catholic University of Korea, Seoul, Republic of Korea, were retrospectively reviewed. Children diagnosed with probable or proven IPA based on the definition of IFD revised by the European Organization for Research and Treatment of Cancer/Invasive Fungal Infections Cooperative Group and the National Institute of Allergy and Infectious Diseases Mycoses Study Group (EORTC-MSG) Consensus Group were enrolled [11]. The follow-up endpoint in the present study was 12 weeks after initiation of antifungal therapy [22], and the enrolled children were divided into survival and fatality groups based on the survival at endpoint. Clinical factors including age; gender; underlying disease and its remission state; administered therapy before the diagnosis of IPA; previous histories of hematopoietic cell transplantation (HCT) and IPA; duration of fever and neutropenia; concurrent infections; and first-line and final antifungal agents were compared between the two groups to determine significant factors associated with 12-week mortality in the early phase of antifungal therapy in IPA patients. Additionally, the results of chest computed tomography (CT) and serum GMI at the initiation and at 1, 2, 3, 4, 6, 8, 10, and 12 weeks after antifungal therapy were also compared between the two groups. This study was approved by the Institutional Review Board of the Seoul St. Mary’s Hospital with an exemption of acquiring informed consent (Approval No.: KC14RISI0791).

### Institutional strategies for antifungal therapy

In our hospital, antifungal prophylaxis and antifungal therapy are performed in accordance with the recommendations of the committee for “Guidelines for the Empirical Therapy of Neutropenic Fever Patients based on Literature in Korea” [23]. Oral fluconazole prophylaxis (3–5 mg/kg/day) was given from the beginning of anti-cancer chemotherapy to the recovery of neutropenia. In the cases receiving HCT, intravenous micafungin prophylaxis (1 mg/kg/day) was given from the beginning of pre-HCT conditioning therapy to engraftment; subsequently, oral fluconazole was administered up until the conclusion of immune suppression. Empirical antifungal therapy was provided if the fever lasted for 3–5 days in children who were expected to have neutropenia of 7 days or longer and who received immune suppressants after allogeneic HCT. Chest CT was performed in children with abnormal findings on chest x-ray or positive serum GMI; pre-emptive antifungal therapy was administered regardless of fever duration if chest CT showed abnormal findings consistent with fungal pneumonia or the serum GMI was positive.

### Galactomannan index measure

GMIs were determined using a Platelia *Aspergillus* EIA kit (BIO-RAD, Marnes-la-Coquette, France) in accordance with the manufacturer’s recommendations. Serum samples showing positive GMI were re-tested for confirmation. Serum GMI tests were performed once or twice a week during neutropenia in children receiving anti-cancer chemotherapy; in children receiving HCT, these tests were performed once or twice a week from pre-HCT conditioning therapy to discharge from the hospital, and then according to the attending physician’s decision after discharge from the hospital. GMI tests for bronchoalveolar lavage (BAL) fluid were performed in addition to serum GMI tests between April 2009 and March 2011 in children who had undergone bronchoscopy.

### Definition

The diagnosis of IPA was based on the patients’ host factor, clinical criteria, and mycological criteria using the definition of IFD revised by the EORTC-MSG Consensus Group in 2008, and IPA status was categorized into possible, probable, and proven IPA [11]. The present study included probable and proven IPA, fulfilling the criteria recommended by the EORTC-MSG Consensus Group. The follow-up endpoint in the present study was defined as 12 weeks from the beginning of parenteral administration of antifungal agents with anti-mold effect [22]. If IPA was diagnosed one or more months after the completion of antifungal therapy for a previous IPA, this was included as a separate IPA case. Fever was defined as a body temperature of 38.0 °C or higher with a tympanic thermometer or 37.5 °C or higher with an axillary thermometer, and neutropenia was defined as an absolute neutrophil count (ANC) less than 500/mm^3^ or an ANC predicted to fall to less than 500/mm^3^ within 2–3 days of fever onset [23]. Chest CT findings were independently determined by two radiologists. The fatality group included all deceased children at follow-up endpoint, since deciding the exact cause of death in immunocompromised patients with IPA is difficult [22]. Positive results of GMI in serum and BAL fluids were defined as >0.5 and >1.0, respectively, based on the 4th European Conference on Infections in Leukemia (ECIL-4) guidelines [12]. Oftentimes, the GMI may rise at some time after antifungal therapy has been initiated for IPA. However, a temporal threshold should be set, and GMI results obtained within this threshold may be considered to be associated with the specific IPA; in contrast, it would be difficult to state with certainty whether the GMI result is related to the specific IPA for results obtained beyond this threshold. In other words, if the GMI tests positive at a prolonged period after an IPA had been suspected and antifungal therapy initiated, then it would be unclear whether the positive GMI is actually related to the initial IPA, or to a different more recent infection. In our study, we set this temporal threshold at 1 month. Hence, only GMI studies performed within 1 month of antifungal therapy for IPA were regarded as related to the specific IPA.

### Statistical analysis

Categorical and numerical factors were compared using a chi-square test and Mann-Whitney test, respectively, when comparing the survival and fatality groups. A multivariate analysis using a binary logistic regression test was performed for significantly different factors in the univariate analysis in order to determine significant factors associated with mortality in IPA patients. The cut-off levels of serum GMI for predicting mortality at each time point during antifungal therapy were determined by using a receiver operating characteristic (ROC) curve. Statistical analysis was performed using SPSS Statistics 17.0 (SPSS Inc., Chicago, IL, USA), and a statistical significance was defined as a two-tailed *p* < 0.05.

## Results

**Fig. 1:**
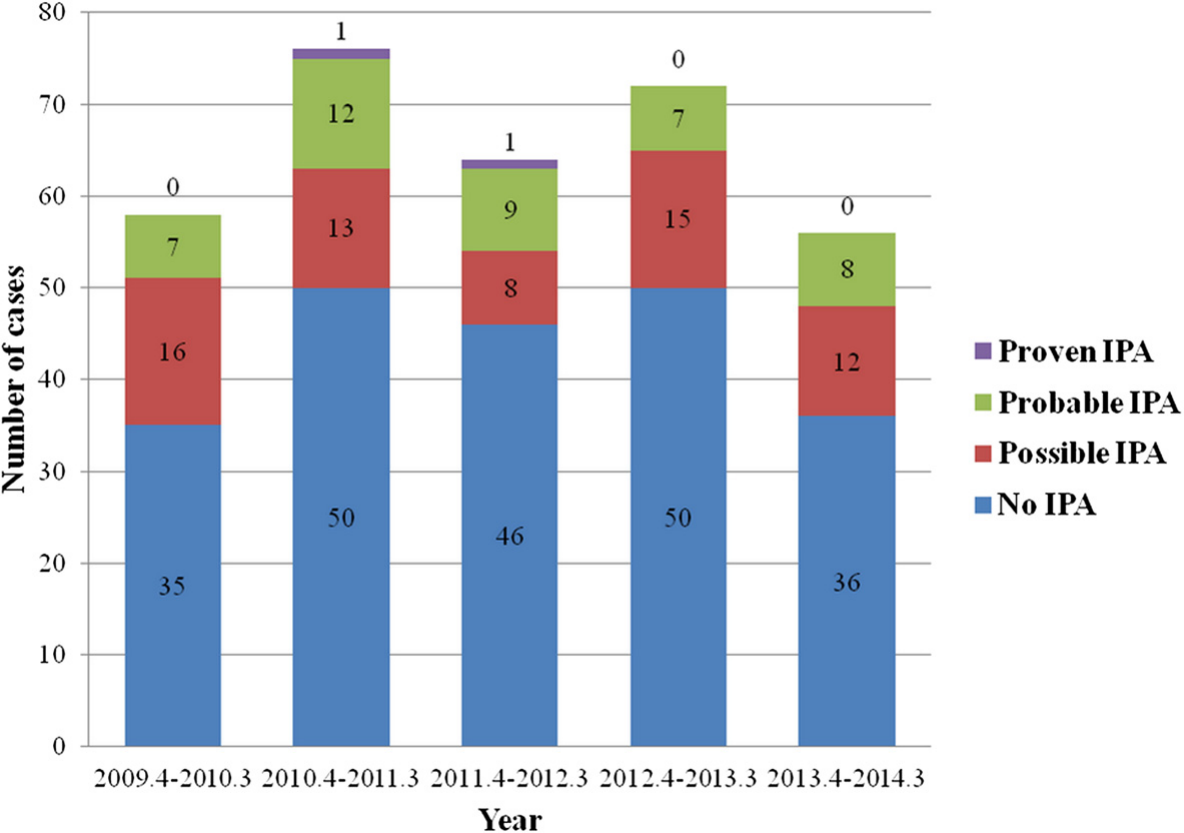
Yearly distribution of the incidence of invasive pulmonary aspergillosis. There was no significant difference for the annual incidence of invasive pulmonary aspergillosis during the study period (*p* = 0.823)

During the study period, 326 cases of parenteral antifungal therapies using anti-mold agents were identified. Among these, 64 cases (19.6 %) of possible IPA, 43 cases (13.2 %) of probable IPA, and 2 cases (0.6 %) of proven IPA were diagnosed. The yearly distribution of probable/proven IPA was not significantly different during the studied 5 years (Fig. 1). Forty-five cases of probable/proven IPA were diagnosed in 41 children, and four children experienced two episodes of IPA each. In these four cases, second episodes of IPA were diagnosed at 3, 4, 14, and 27 months after the completion of antifungal therapy for the first IPA. In another child, the first IPA was diagnosed before the study period and the second episode of IPA was diagnosed once the study had begun, 6 weeks after the completion of antifungal therapy for the first IPA. Mean age of the enrolled 45 cases of probable/proven IPA was 11.3 ± 5.1 years, and 30 patients (66.7 %) were males. Fifteen (33.3 %) died within 12 weeks of antifungal therapy.

### Comparison of clinical characteristics between the survival and fatality groups

Clinical characteristics were compared between the survival and fatality groups in order to find significant factors predicting mortality in IPA patients in the early phase of antifungal therapy (Table 1). Significantly more cases were male in the fatality compared with the survival group (*p* = 0.044), however, there were no significant differences in mean age and the type of underlying hematologic/oncologic diseases between the two groups. Significantly more cases in the fatality group were not in complete remission (CR) of the underlying hematologic malignancies compared with the survival group (*p* = 0.016), and therefore, the frequency of re-induction/salvage and palliative chemotherapy for underlying diseases was significantly higher in the fatality group than in the survival group (*p* = 0.035). However, none of these factors showed a significant relationship with mortality in a multivariate analysis (Table 1). Halo sign was the most common finding on chest CT in both groups, and serum GMIs were positive in 76.7 % of the survival group and 93.3 % of the fatality group (Table 1). GMIs for BAL fluid were tested in 11 cases (10 in the survival group and one in the fatality group), and were positive in six cases (54.5 %) in the survival group. Only one (16.7 %) of the six cases with positive BAL fluid GMI showed positive serum GMI. *Aspergillus* species were identified in BAL fluid cultures in two cases (18.2 %) of the survival group, and both of these cases showed negative BAL fluid GMI. Two children diagnosed with proven IPA were included in the survival group; pulmonary lobectomy was performed in both of these cases, and *Aspergillus* species were proven histopathologically in the excised lung tissue.

**Table 1:** Comparison of characteristics between the survival and fatality groups

Factor	Survival group (*n* = 30)	Fatality group (*n* = 15)	*p-*value (univariate)	*p-*value (multivariate)
Gender			0.044	
Male	17 (56.7)	13 (86.7)		
Female	13 (43.3)	2 (13.3)		0.104
Age (years)	13 (4-19)	11 (1-19)	0.111	
Underlying disease			0.296	
Acute myeloid leukemia	12 (40.0)	7 (46.7)		
Acute lymphoblastic leukemia	13 (43.3)	4 (26.7)		
Severe aplastic anemia	5 (16.7)	2 (13.3)		
Undifferentiated acute leukemia	0 (0.0)	1 (6.7)		
Non-Hodgkin lymphoma	0 (0.0)	1 (6.7)		
Remission status of the underlying disease^a^			0.016	
Complete remission status	9 (36.0)	0 (0.0)		
Not complete remission status	16 (64.0)	13 (100.0)		1.000
Type of preceding chemotherapy			0.035	
No chemotherapy	6 (20.0)	1 (6.7)		
Induction	4 (13.3)	0 (0.0)		1.000
Re-induction or salvage	9 (30.0)	9 (60.0)		0.999
Consolidation	4 (13.3)	0 (0.0)		1.000
Maintenance	1 (3.3)	0 (0.0)		1.000
Palliative	1 (3.3)	4 (26.7)		0.999
Allogeneic hematopoietic cell transplantation	5 (16.7)	1 (6.7)		1.000
Previous history of hematopoietic cell transplantation	14 (46.7)	8 (53.3)	0.673	
Previous history of invasive aspergillosis	4 (13.3)	1 (6.7)	0.651	
Duration of fever (days)	2 (0–10)	3 (0–17)	0.419	
Duration of neutropenia at the beginning of antifungal therapy			1.000	
≤2 weeks	14 (46.7)	7 (46.7)		
>2 weeks	16 (53.3)	8 (53.3)		
Presence of other accompanying infections	11 (36.7)	4 (26.7)	0.502	
Chest computed tomography findings				
Consolidations with a halo sign	21 (70.0)	14 (93.3)	0.129	
Consolidations without a halo sign	9 (30.0)	1 (6.7)	0.129	
Air-crescent sign	0 (0.0)	0 (0.0)	NA	
Cavity	1 (3.3)	1 (6.7)	1.000	
Positive serum galactomannan results	23 (76.7)	14 (93.3)	0.234	

Data are median (range) or No. (%) of cases

*NA* not available

^a^The remission status was determined in 38 children (25 in the survival group and 13 in the fatality group), except for children with severe aplastic anemia

**Fig. 2:**
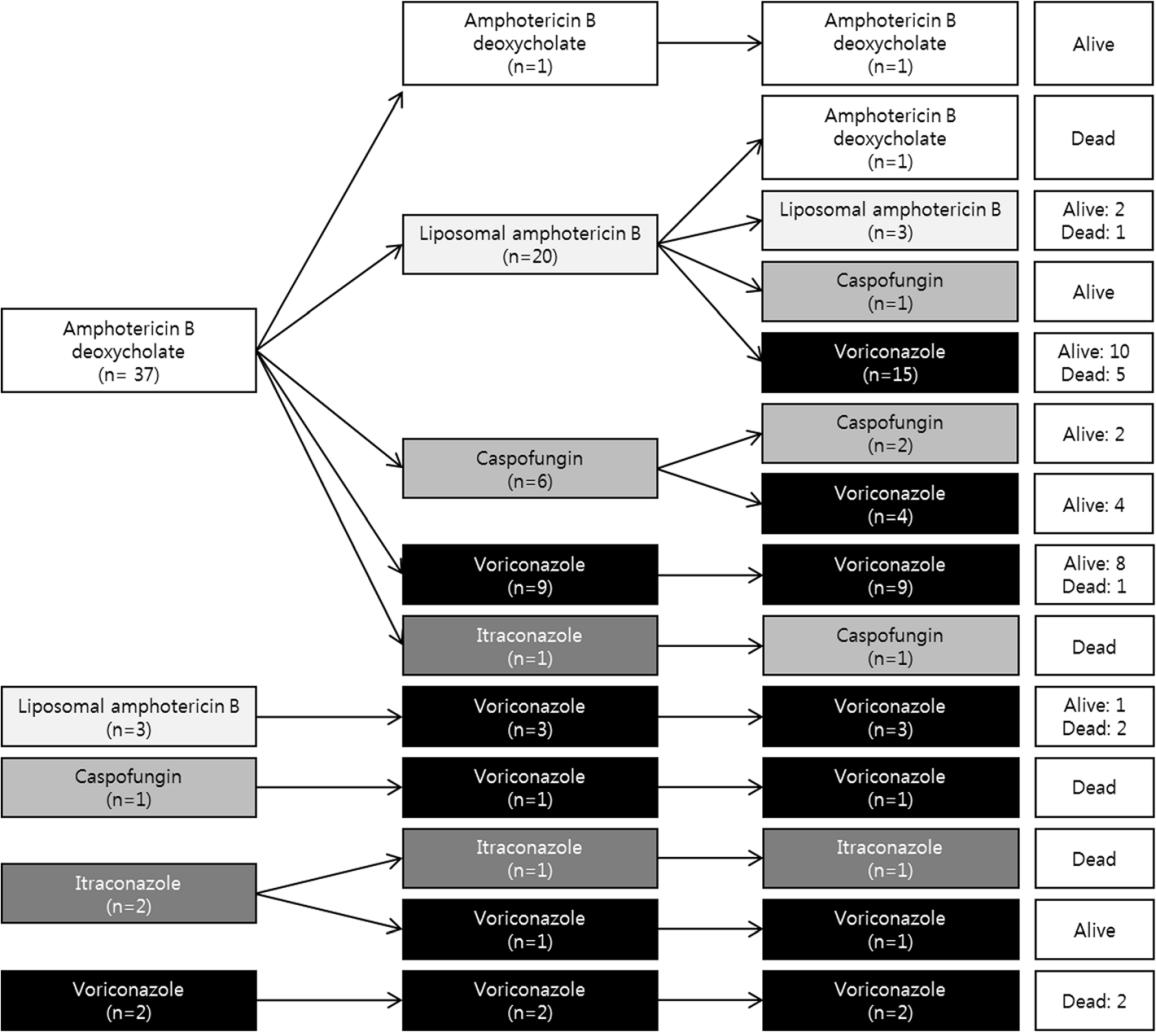
Diagram for antifungal therapy. Amphotericin B deoxycholate was the most commonly administered first-line antifungal agent (82.2 %). However, most of the first-line agents were changed to other agents, and voriconazole was eventually administered in 77.8 % of the cases

Thirty-one cases (68.9 %) had received anti-fungal prophylaxis, and most of them (83.9 %) had received oral fluconazole (Table 2). Amphotericin B deoxycholate was administered as a first-line antifungal agent in 82.2 % of all probable/proven IPA cases, and first-line agents were changed to other antifungal agents in 41 cases (91.1 %) a median of 4 days (range: 0–34) after the initiation of antifungal therapy (Fig. 2). Finally, 35 cases (77.8 %) completed antifungal therapy with oral or intravenous voriconazole. None of the patients received antifungal combination therapy. The administration of first-line and final antifungal agents was not significantly different between the survival and fatality groups (Table 2). Further, the frequency of receiving voriconazole and the time of voriconazole therapy initiation were not significantly different between the two groups (data not shown). In the fatality group, the duration of fever was significantly longer (*p* = 0.031) during antifungal therapy and more patients experienced neutropenia lasting longer than 2 weeks (*p* = 0.033) after antifungal therapy compared with the survival group (Table 2).

**Table 2:** Antibacterial and antifungal therapy for children with probable/proven invasive pulmonary aspergillosis

Factor	Survival group (*n* = 30)	Fatality group (*n* = 15)	*p-*value
Antibacterial therapy on the diagnosis of IPA			0.511
Meropenem with teicoplanin	15 (50.0)	10 (66.7)	
Meropenem	7 (23.3)	2 (13.3)	
Piperacillin/tazobactam with isepamicin	4 (13.3)	3 (20.0)	
Cefepime	1 (3.3)	0 (0.0)	
None	3 (10.0)	0 (0.0)	
Anti-fungal prophylaxis			0.327
No prophylaxis	11 (36.7)	3 (20.0)	
Oral fluconazole	15 (50.0)	11 (73.3)	
Oral itraconazole or intravenous micafungin	4 (13.3)	1 (6.7)	
First-line antifungal agents			
Amphotericin B deoxycholate	28 (93.3)	9 (60.0)	0.059
Liposomal amphotericin B	1 (3.3)	2 (13.3)	
Caspofungin	0 (0.0)	1 (6.7)	
Itraconazole	1 (3.3)	1 (6.7)	
Intravenous voriconazole	0 (0.0)	2 (13.3)	
Final antifungal agents			0.657
Amphotericin B deoxycholate	1 (3.3)	1 (6.7)	
Liposomal amphotericin B	2 (6.7)	1 (6.7)	
Caspofungin	3 (10.0)	1 (6.7)	
Itraconazole (oral)	0 (0.0)	1 (6.7)	
Voriconazole (intravenous or oral)	24 (80.0)	11 (73.3)	
Total duration of fever (days)	7 (1–32)	17 (1–64)	0.031
Duration of neutropenia after antifungal therapy			0.006
≤2 weeks	21 (70.0)	4 (26.7)	
>2 weeks	9 (30.0)	11 (73.3)	

Data are median (range) or No. (%) of cases

*IPA* invasive pulmonary aspergillosis

### Comparison of serum galactomannan index between the survival and fatality groups

Serum GMI was positive in 37 (82.2 %) cases. Although piperacillin/tazobactam may cause false positive results for serum GMI, piperacillin/tazobactam was being administered to only seven (15.5 %) children on the diagnosis of IPA and the frequencies of receiving piperacillin/tazobactam on the diagnosis of IPA were not significantly different between cases with and without positive serum GMI (16.2 % vs. 12.5 %, *p* = 1.000). Eight (21.6 %) of the 37 cases with positive serum GMI had received piperacillin/tazobactam within 3 days before positive serum GMI results were identified. On the other hand, four (50.0 %) of the eight cases with negative serum GMI had received piperacillin/tazobactam during repeated serum GMI tests.

**Fig. 3:**
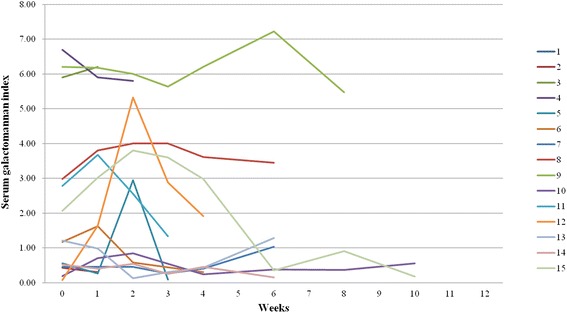
Trends of serum galactomannan indices during antifungal therapy in the fatality group

Serum GMIs from the initiation of antifungal therapy to 12 weeks after antifungal therapy were compared between the survival and fatality groups. Serum GMIs were higher in the fatality group than in the survival group during the entire period of follow-up, and the GMIs on the initiation of antifungal therapy and at 1, 2, 6, and 8 weeks after antifungal therapy showed significant differences between the two groups (Table 3). The frequency of positive GMI was also higher in the fatality group than in the survival group during the entire period of follow-up with significant differences at 1, 2, and 6 weeks after antifungal therapy. The differences in serum GMIs at 6 and 8 weeks after antifungal therapy were not considered clinically significant because nine cases (60.0 %) in the fatality group had died within 6 weeks of receiving antifungal therapy. The cut-off levels of serum GMI for predicting mortality at the remaining time points were determined by using an ROC curve; the respective areas under the curve at the initiation of antifungal therapy and 1 and 2 weeks after antifungal therapy were 0.723, 0.812, and 0.795, respectively. As a result, the serum GMI at 1 week after antifungal therapy was most significantly associated with mortality, and its cut-off value predicting mortality was 1.5 with a sensitivity of 61.5 %, specificity of 89.3 %, positive predictive value (PPV) of 72.7 %, and negative predictive value (NPV) of 83.3 %. The trends of serum GMI levels in the fatality group are shown in Fig. 3.

**Table 3:** Comparison of serum galactomannan indices between the survival and fatality groups during antifungal therapy

	Time	Initial (*n* = 30/15)	1 week (*n* = 28/13)	2 weeks (*n* = 25/12)	3 weeks (*n* = 20/9)	4 weeks (*n* = 15/9)	6 weeks (*n* = 15/7)	8 weeks (*n* = 14/3)	10 weeks (*n* = 11/2)	12 weeks (*n* = 17/0)
GMI, median (range)	Survival group (*n* = 30)	0.46 (0.15–6.19)	0.39 (0.09–1.93)	0.38 (0.11–2.58)	0.33 (0.11–1.45)	0.27 (0.18–1.81)	0.21 (0.07–0.74)	0.27 (0.08–2.43)	0.17 (0.09–2.74)	0.24 (0.09–6.98)
	Fatality group (*n* = 15)	1.21 (0.08–6.70)	1.64 (0.27–6.20)	2.76 (0.13–6.00)	1.34 (0.09–5.64)	0.46 (0.24–6.21)	1.04 (0.15–7.23)	0.91 (0.37–5.48)	0.37 (0.18–0.56)	NA
	*p*-value	0.015	0.001	0.004	0.164	0.060	0.009	0.032	0.553	NA
GMI positivity	Survival group (*n* = 30)	13 (43.3)	12 (42.9)	10 (40.0)	7 (35.0)	5 (33.3)	1 (6.7)	2 (14.3)	3 (27.3)	2 (11.8)
	Fatality group (*n* = 15)	11 (73.3)	10 (76.9)	10 (83.3)	5 (55.6)	4 (44.4)	4 (57.1)	2 (66.7)	1 (50.0)	NA
	*p*-value	0.057	0.042	0.013	0.422	0.678	0.021	0.121	1.000	NA

*GMI* galactomannan index, *NA* not available

## Discussion

In the present study, risk factors for mortality in immunocompromised children with IPA were investigated. Male gender, neutropenia on IPA diagnosis, acute myeloid leukemia rather than acute lymphoblastic leukemia as an underlying hematologic malignancy, uncontrolled underlying malignancy, disseminated infection, accompanying renal dysfunction, receiving allogeneic HCT, and pleural effusion or micronodules in chest CT had previously been reported as risk factors for mortality in patients with IPA [3, 10, 24–28]. Male gender, non-CR state of underlying hematologic malignancies, and receiving re-induction/salvage or palliative chemotherapy were risk factors for mortality in a univariate analysis in the present study. However, none of them were significantly associated with mortality in a multivariate analysis. Although the duration of fever and neutropenia was significantly longer in the fatality group compared with the survival group in the present study, mortality cannot be predicted by using these factors in the early phase of antifungal therapy. The presence of halo signs in chest CT has previously been reported as a favorable factor for outcome in IPA patients [29]. However, this was not the case in the present study. Chest CT halo, which is considered an early radiologic finding of IPA [30, 31], was observed in 93.3 % of the cases in the fatality group in the present study; therefore, this represented an early diagnosis of IPA in the children enrolled in the present study, even in the fatality group. Accordingly, in the present study, mortality would be expected to be affected by the status of the patients’ underlying diseases and the degree of response to antifungal therapy rather than by a delayed diagnosis of IPA. In addition, the fact that there were no significant differences in first-line and final antifungal agents between the survival and fatality groups shows that a differentiated and more intensive antifungal therapy was not administered to children with IPA at a high risk for mortality.

The association between a high serum GMI on the diagnosis of IPA and poor outcomes and increased mortality in IPA patients has been previously reported [18, 32]. Rohrlich *et al.* demonstrated that serum GMI is useful for diagnosing IPA in children, and reported a relationship between high serum GMI on the diagnosis of IPA and increased mortality [21]. IPA patients with persistently positive serum GMI levels during antifungal therapy also showed higher mortality compared with those with negative conversion of serum GMI [14–17]. In addition, the degree of reduction of serum GMI within 1 week after the diagnosis of IPA was reported to be related to mortality [20, 33], and the increase of serum GMI by one or more in 1 or 2 weeks after the diagnosis of IPA was reported to be related to poor prognosis in IPA patients, too [19]. In the present study, serum GMI 1 week after antifungal therapy exhibited the most significant relationship with mortality, and a significantly higher number of cases in the fatality group showed positive GMI results compared with those in the survival group at that time. In particular, serum GMI of 1.5 or higher at 1 week after antifungal therapy exhibited 72.7 % of PPV predicting mortality within 12 weeks of antifungal therapy. If we put together the result of the present study and previously reported results, reduction of serum GMI in the early phase of antifungal therapy, especially in 1 or 2 weeks of antifungal therapy, should be associated with improving prognosis in IPA patients. The level of serum GMI was significantly associated with the degree of fungal burden of *Aspergillus* species in the lung, and appropriate antifungal therapy decreased serum GMI levels and increased survival rate in animal models [34–36]. Persistently positive serum GMI despite antifungal therapy represents persisting fungal burden and continuous fungal infection. This could be caused by more suppressed immunity of the fatality group compared with the survival group, considering more children in the fatality group received palliative care for uncontrolled underlying malignancies and experienced prolonged neutropenia. Also, this may mean inadequacy of the administered antifungal therapy. In particular, the association between mortality and the trend of serum GMIs in the early phase of antifungal therapy suggests the importance of administering appropriate first-line antifungal agents. Therefore, empirical antifungal therapy using the most effective antifungal agent against IPA in immunocompromised patients should be necessary in order to improve outcomes in those patients.

Voriconazole showed significant reduction of mortality in IPA patients compared with previously used amphotericin B deoxycholate [8], and it is currently recommended as the drug of choice for IPA [12, 37]. However, voriconazole therapy has still exhibited a mortality of 30 %. For IPA patients at high risk of mortality, who show a high serum GMI after 1 week of antifungal therapy, more intensive antifungal therapy, such as antifungal combination and higher dose of antifungal agents, may be considered in order to improve outcomes. Several studies showed an increased survival rate in children with IA treated with voriconazole or liposomal amphotericin B in combination with caspofungin compared with its monotherapy, other studies showed that the efficacy and safety were not significantly improved with antifungal combination therapy; further, prospective controlled studies on antifungal combination therapy in children have not been published [38]. However, a recently reported randomized trial showed a lower 6-week mortality in adults with IPA receiving voriconazole and anidulafungin compared with those receiving voriconazole monotherapy, although the difference was not statistically significant [39]. High dose liposomal amphotericin B (10 mg/kg/day) did not induce a significant reduction of mortality compared to 3 mg/kg/day of liposomal amphotericin B monotherapy or in combination with caspofungin [40, 41]. High dose voriconazole therapy seems not to be useful because it has a narrow therapeutic window and its adverse effects are associated with a high serum drug level [42]. However, a higher dose of voriconazole is usually necessary in children than in adults [37]; therefore, appropriate dose adjustment of voriconazole under close therapeutic drug monitoring (TDM) should be performed to achieve proper therapeutic efficacy, especially in children with IPA. Further studies should be conducted in the future to determine measures for improving the therapeutic efficacy of antifungal therapy.

This study has several limitations arising from its retrospective nature. GMI tests for BAL fluid samples were performed only during the first 2 years of the study period because of restriction from the government regulations of the Korean National Health Assurance system. Some patients with a negative serum GMI might test positive for BAL fluid GMI, and these might not be considered in the last 3 years of the study. Piperacillin/tazobactam and isepamicin have been administered as empirical antibiotics for febrile neutropenia in our hospital, and therefore, some children might show falsely positive serum GMI. In the present study, 21.6 % of the cases with positive serum GMI received piperacillin/tazobactam within 3 days before identifying positive serum GMI results, and 50.0 % of the cases without positive serum GMI had received piperacillin/tazobactam during repeated serum GMI tests. The frequency of receiving piperacillin/tazobactam on the diagnosis of IPA was not significantly different between the cases with and without positive serum GMI. The effect of falsely positive serum GMI due to the administration of piperacillin/tazobactam may be negligible in the present study. Most cases in the present study received amphotericin B deoxycholate as a first-line antifungal agent due to the government regulation based on the National Health Assurance policy. In other institutions using antifungal agents other than amphotericin B deoxycholate as a first-line antifungal drug, the results of the present study may not be applicable. However, Koo *et al.* reported that the early decrease in serum GMI was associated with reduced mortality when most patients received caspofungin or voriconazole therapy [33].

## Conclusions

In the present study, the outcome of immunocompromised children with IPA was significantly associated with the early trend of serum GMIs during antifungal therapy. Therefore, empirically administered antifungal agents in immunocompromised patients at risk of IPA should be fully effective against IPA in order to improve their clinical outcome. In addition, IPA patients at high risk for mortality, who show a persistently high level of serum GMI within 1 week of antifungal therapy, should receive more intensive antifungal therapy.
